# Hydrogen Peroxide and Amyotrophic Lateral Sclerosis: From Biochemistry to Pathophysiology

**DOI:** 10.3390/antiox11010052

**Published:** 2021-12-27

**Authors:** Nitesh Sanghai, Geoffrey K. Tranmer

**Affiliations:** 1 College of Pharmacy, Rady Faculty of Health Science, University of Manitoba, Winnipeg, MB R3E 0T5, Canada; geoffrey.tranmer@umanitoba.ca; 2 Department of Chemistry, Faculty of Science, University of Manitoba, Winnipeg, MB R3T 2N2, Canada

**Keywords:** motor neurodegenerative disease, amyotrophic lateral sclerosis, superoxide dismutase 1, mutant superoxide dismutase 1, reactive oxygen species, hydrogen peroxide, misfolding, aggregation, fibrilization, redox dyshomeostasis, TAR-DNA binding protein 43, neuronal cytoplasmic inclusions

## Abstract

Free radicals are unstable chemical reactive species produced during Redox dyshomeostasis (RDH) inside living cells and are implicated in the pathogenesis of various neurodegenerative diseases. One of the most complicated and life-threatening motor neurodegenerative diseases (MND) is amyotrophic lateral sclerosis (ALS) because of the poor understanding of its pathophysiology and absence of an effective treatment for its cure. During the last 25 years, researchers around the globe have focused their interest on copper/zinc superoxide dismutase (Cu/Zn SOD, SOD1) protein after the landmark discovery of mutant SOD1 (mSOD1) gene as a risk factor for ALS. Substantial evidence suggests that toxic gain of function due to redox disturbance caused by reactive oxygen species (ROS) changes the biophysical properties of native SOD1 protein thus, instigating its fibrillization and misfolding. These abnormal misfolding aggregates or inclusions of SOD1 play a role in the pathogenesis of both forms of ALS, i.e., Sporadic ALS (sALS) and familial ALS (fALS). However, what leads to a decrease in the stability and misfolding of SOD1 is still in question and our scientific knowledge is scarce. A large number of studies have been conducted in this area to explore the biochemical mechanistic pathway of SOD1 aggregation. Several studies, over the past two decades, have shown that the SOD1-catalyzed biochemical reaction product hydrogen peroxide (H_2_O_2_) at a pathological concentration act as a substrate to trigger the misfolding trajectories and toxicity of SOD1 in the pathogenesis of ALS. These toxic aggregates of SOD1 also cause aberrant localization of TAR-DNA binding protein 43 (TDP-43), which is characteristic of neuronal cytoplasmic inclusions (NCI) found in ALS. Here in this review, we present the evidence implicating the pivotal role of H_2_O_2_ in modulating the toxicity of SOD1 in the pathophysiology of the incurable and highly complex disease ALS. Also, highlighting the role of H_2_O_2_ in ALS, we believe will encourage scientists to target pathological concentrations of H_2_O_2_ thereby halting the misfolding of SOD1.

## 1. Introduction

Free radicals, in the context of living cells, are bio-reactive species. They are unstable, short-lived, and highly reactive chemical species. They react with various biological molecules to complete the quest for their electrons to become stable [1]. Free radicals have implications in diverse kinds of neurodegenerative diseases like ALS [2], Alzheimer’s disease (AD) [3], Parkinson’s disease (PD) [4], Huntington’s disease(HD) [5]. ALS is one of the most devastating and highly complex neurodegenerative diseases. Disease progression is usually rapid, and the average survival is only 2–3 years from symptom onset. Despite intense research, the cause of the disease is still unknown, and the etiology of motor neurodegeneration (MND) is still incompletely understood. The development of new therapies is hindered, because of the poor understanding of the molecular mechanism and biological process associated with the disease. ALS is a multifactorial disease, with multiple genetic and environmental factors playing an important role in disease pathology [6]. Out of all the ALS cases known, around 20% of familial MND cases (5–10% of all cases are familial) are caused by mutations in a ubiquitous metalloenzyme called SOD1 [7]. The other 90% of cases are sporadic in nature and termed sALS. The discovery of mutations in the superoxide dismutase 1 (SOD1) gene that encodes antioxidant SOD1 enzyme, made it possible to understand the progression of the disease. Substantial evidence has shown that abnormal conformational change in the structure of SOD1 protein causes misfolding and aggregation, and thus is thought to have neurotoxic properties, which are observed in both forms of ALS [8,9,10]. It’s has been more than two decades after the landmark discovery of mutant SOD1 (mSOD1) [7], and scientists are still trying to untangle the mystery behind the misfolding of SOD1. A large body of evidence has shown that redox dysregulation of SOD1 during oxidative stress is largely involved in changing the biochemical and biophysical properties of SOD1 causing its abnormal aggregation and misfolding [11,12,13,14,15,16], which is evident in the cellular toxicity observed in ALS [17,18,19,20]. Studies have shown that hydrogen peroxide (H_2_O_2_) modulates the redox state of SOD1, controlling the folding and misfolding of SOD1. The abnormal conformational kinetic change in the SOD1 structure is due to the increased concentration of H_2_O_2_, culminating in motor neuron death in ALS [21,22,23,24,25]. Furthermore, toxic SOD1 has been found to modulate TDP-43 localization from the cytoplasm to the nucleus. Abnormal oligomerization of SOD1 causes mislocalization of TDP-43 leading to unusual accumulation in the cytosol, thus causing the gain of a new pathogenic function observed in the degeneration of motor neurons of ALS [21,26,27,28]. Herein, we explore the role of H_2_O_2_ as a major determinant in the pathophysiology of ALS.

## 2. Bio-Reactive Oxygen Species in Living Cells

**Figure 1:**
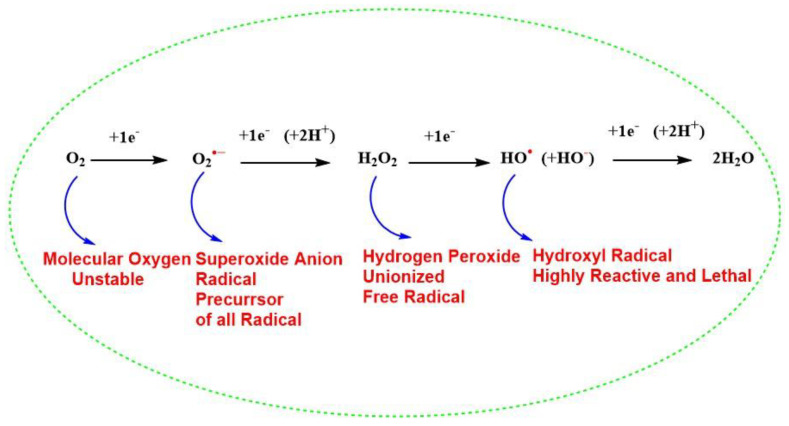
Successive four-electron reduction of molecular oxygen to generate water and different reactive oxygen species during cellular metabolism.

Free radicals are chemically reactive species, having an electrophilic nature, Refs. [29,30] as a result of an unpaired electron in their outermost orbital. Because of these unpaired electrons, they are highly unstable and reactive and are continuously produced in vivo [31]. They also act as a vital molecule for the existence of life on earth and regulate the metabolism of cells and take part in the generation of ATP for the survival of cells [31,32,33]. Although it is important for life, molecular oxygen (O_2_, also termed triplet oxygen) is a biradical and unstable molecule with two unpaired electrons with parallel spins in the same direction in its outer orbital and thus paramagnetic character [34]. The presence of two unpaired electrons with unpaired spin and high resonance stabilization energy of the **π** electron [34] decreases the reactivity of (O_2_) showing its dynamic “quantum quirkiness” [35] with other biomolecules of life, which has a normal configuration with a paired electron in the outermost orbital with opposite spins. Thus, the stable nonradical molecule must undergo spin inversion to donate an electron to molecular oxygen, which is restricted and against the law of quantum mechanics [32]. To overcome this chemical barrier, oxygen molecule reacts with paramagnetic transition metals like iron (Fe) and copper (Cu), which are found primarily in biomolecules or there is successive addition of electrons to the molecular oxygen during cellular metabolism. The reduction of univalent molecular oxygen mainly takes place in mitochondria with the help of cytochrome c oxidase (Complex IV), during oxidative phosphorylation and leads to the addition of four electrons to O_2_ to reduce it to H_2_O (Figure 1). This means that O_2_ is ultimately reduced to water (H_2_O) by producing the intermediate radicals, such as superoxide anion radical (O_2_^•−^), H_2_O_2,_ and hydroxyl radical (HO^•^). Out of these (O_2_^•−^) and HO^•^ are highly reactive free radicals and react with organic biomolecules, leading to the production of other intermediates and thus produce free radicals, creating oxidative stress and ROS inside living cells, disrupting the normal redox biology of living cells [36,37]. In addition, Coenzyme Q (CoQ) is an essential, lipid-soluble component of the mitochondrial membrane electron transport chain (ETC) and, it has been reported that the semiquinone radical generated from human antioxidant coenzyme Q (Q10) can lead to an increase in (O_2_^•−^) in humans [38,39], due to the abnormal dysfunction of mitochondrial ETC. This change in bioenergetics of mitochondrial ETC leads to higher production of (O_2_^•−^) in cells [40,41]. Biomolecules like membrane phospholipids [42], mitochondrial DNA [43], proteins, peptides, sulfur-containing amino acids like methionine (Met), and cysteine (Cys) contain highly nucleophilic reactive functional groups, such as sulfhydryl or thiols (SH) are vulnerable to be oxidized by excessive ROS [44]. For instance, SOD1 protein undergoes misfolding due to a conformational change caused by the excessive irreversible oxidation of (SH) group to sulfinic acid (-SO_2_H)and sulfonic acid (-SO_3_H) modifications however, the first step in the oxidation of the (SH) group to sulphenic acid (-SOH) remains reversible and gives rise to the formation of a disulfide bond (-S-S-) [45]. Therefore, the balance between oxidation-reduction (redox) processes, is an evolutionarily conserved process in the biological system and is maintained by the presence of efficient reductase enzymes. Further, the (-S-S-) can be salvaged for SOD1 by the presence of efficient reductants. However, under various cellular stress condition, oxidative stress cause abnormal oxidative PTMs and leads to change in protein biochemistry as indicated. Thus, the living cells maintain a redox balance between biological natural reductants and oxidants to maintain homeostasis inside the living cells for their survival [46,47,48].

‘Oxidative stress’ is a term associated with both enhanced production of ROS and reduced efficacy of protection by antioxidant enzymes or reduced molecular weight antioxidants. After the discovery of nitric oxide radical (NO^•^) as a biological signaling molecule [49], it was unraveled that peroxynitrite (ONOO^−^) may be formed in vivo from superoxide and nitric oxide to create ‘nitrosative stress’ and reactive nitrogen species(RNS) [50]. Tyrosine is a common target of nitrosative stress and 3-nitrotyrosine (3-NT) has been detected in various neurodegenerative diseases [51], such as ALS [52], AD [51], and PD [53].

## 3. The Chemistry of Hydrogen Peroxide

H_2_O_2_ is the nonionized 2-electron reduction product of unstable molecular oxygen [54], which plays a central role in maintaining the redox cycle of living cells [55]. H_2_O_2_ is a diverse potent oxidizing and inexpensive chemical molecule, which has chemical applications [56], biological functions [55], and therapeutic applications which include antimicrobial and oxidizing agents [57]. Over the last decade, H_2_O_2_ has been used as a green oxidant and alternate source of oxygen to convert biomass to chemical synthesis [56]. It also acts as an environmentally friendly oxidizing catalyst in many oxidizing chemical processes because the end product of its decomposition is only a water molecule [58]. H_2_O_2_ is one of the closest cousins of water and is a non-planar molecule having an open book structure. It is also regarded as the smallest chiral molecule, which can undergo a disproportionation reaction to act both as oxidizing and reducing agent [59,60].

H_2_O_2_ is a strong oxidant, having a reduction potential of 1.76 V at pH 7.0, 25 °C. Therefore, it is more oxidizing than hypochlorous acid (HOCl) or (ONOO^−^), for which the reduction potentials are 1.48 and 1.4 V, respectively. However, relative to these two reactive species, the reactivity of H_2_O_2_ is relatively low with various biological molecules like nucleic acid, proteins, and lipids. Further, this can be explained by its higher activation energy barrier, which must be overcome to release its oxidizing power. In other words, the chemical reactions of hydrogen peroxide are kinetically controlled rather than thermodynamically driven [61]. The H_2_O_2_ produced during cellular metabolism is found to be stable, compared to (HOCl) having a half-life in minutes and (ONOO^−^) having a half-life of 10^−3^ s [1,62]. It is a major oxidant produced by the activated neutrophils at the site of inflammation from H_2_O_2_ and chloride, catalyzed by the enzyme myeloperoxidase, a leukocyte-derived enzyme. HOCL exerts its oxidizing power through the oxidation and chlorination of biomolecules like nucleic acids, lipids, and cholesterol [63]. It confers its oxidizing power via chlorination of amino acids like tyrosine to form 3-chlorotyrosine and damage the collagen [64]. HOCl is more reactive than H_2_O_2_ (rate constants of 3 × 10^7^ M^−1^ s^−1^ and 0.9 M^−1^ s^−1^, respectively), however, the redox potential for the 2-electron reduction is larger for the H_2_O_2_ in forming H_2_O, than for the former in releasing chloride [65]. ONOO^−^ is formed by the reaction between O_2_^•−^ and NO^•^. It is highly toxic to biomolecules, oxidizes lipids, Met, and tyrosine residues in proteins [66]. The nitrotyrosine residues are considered as a marker of ONOO^−^ induced cellular damage, potentially serving as a “peroxynitrite footprint” in the biological oxidation process. The main biological target of ONOO^−^ is DNA, where it oxidizes the base pairs, causing nitrative and oxidative DNA lesions such as 8-nitroguanine and 8-oxodeoxyguanosine respectively [67,68]. Recent evidence has shown that the protein (SH) significantly reacts faster with ONOO^−^ compared to H_2_O_2_ [69,70].

H_2_O_2_ is a weak one-electron oxidant, although the one-electron reduction product, the HO^•^ is one of nature’s most vulnerable bio-reactive species, which can create an oxidizing environment in the living cell and ultimately lead to its death [71,72,73]. The redox property of H_2_O_2_ is dependent upon the pH of the solutions, as the pKa of H_2_O_2_ is 11.6, so it is mostly unionized at physiological pH. The strong nucleophilicity of hydroperoxide nucleophile (^−^OOH) is limited due to its high pKa. H_2_O_2_ also acts as electrophile due to the polarization of the peroxide bond (O−O). The homolytic bond dissociation enthalpies of peroxide in H_2_O_2_ at 298 K is 50 kcal/mol, whereas the heterolytic bond dissociation enthalpies of H_2_O_2_ is 290 kcal/mol. The unexpected chemical reactivity of hydrogen peroxide is generally attributed to the weakness of (O-O) and therefore, it is homolytically cleaved in presence of heating, radiolysis, photolysis, or transition metals [74]. Prof. John O. Edwards, a pioneer in the field of peroxide chemistry, has demonstrated the mechanism of peroxide chemistry exquisitely. According to him, thiolates (-S^−^) are more reactive than (SH). Hence, (-S^−^), being nucleophilic, react with H_2_O_2_, an electrophile, by bimolecular nucleophilic substitution (SN2) reaction mechanism displacing ^−^OH as leaving group [75]. However, recent developments on this mechanism suggest that the by-product of the reaction is H_2_O instead of hydroxyl anion (HO^−^) [76,77]. The nucleophilic reaction of a protein (-S^−^) on electrophilic H_2_O_2_ forms H_2_O as a by-product and results in the formation of cysteine sulphenic acid (CysSOH), the process is also known as S-sulfenylation. Depending upon the protein microenvironment where the thiolate is located, the reaction is exclusive to (-S^−^), and at physiological pH, dependent upon the pKa values of the sulfur-containing amino acids. In addition, hydrogen bonding also plays an important part in the ionization of (SH) to (-S^−^) [78,79,80]. Further, the SN2 paradigm was also challenged and proved by hybrid quantum-classical (QM-MM) molecular dynamics simulations [79]. The nucleophilicity of H_2_O_2_ could be explained by the reaction of ^−^OOH with organohalides [81], and oxidation of boron compounds [82]. We can thus conclude that H_2_O_2_, a green redox molecule that has properties of both oxidant and reductant, also can be a nucleophile and electrophile in chemical reactions. Having all these characteristics it reacts poorly with most biological molecules because of the high activation energy barrier and the reaction rate is kinetically controlled. Thus, the vigorous oxidizing power of H_2_O_2_ comes indirectly from its transition metal catalysis into HO^•^ by Fenton and Haber-Weiss reactions [61]. In addition, Cu/Zn SOD1, which is a ubiquitous natural antioxidant enzyme, is mainly involved in dismutation of ionizable toxic O_2_^•−^ radical in vivo to produce a non-ionizable less toxic redox molecule H_2_O_2_, which has the ability to generate OH^•^ free radical, thereby acting as pro-oxidant in certain disease conditions [23,83].

### 3.1. Hydrogen Peroxide as Double Edge Sword in Living Cells

**Figure 2:**
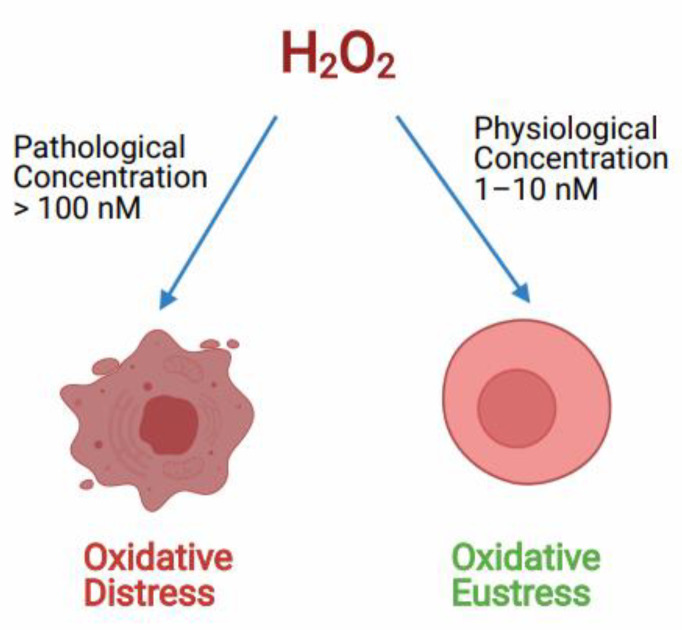
Role of H_2_O_2_ controlling the oxidative redox balance of the cell at different concentrations. Hydrogen peroxide (H_2_O_2_) controls the cell signaling process at physiological concentration called oxidative Eustress. Whereas, at pathological concentration, H_2_O_2_ causes cell death due to oxidative distress.

H_2_O_2_acts both as a redox signaling molecule and an oxidative stress molecule. As a signal transduction molecule H_2_O_2_ has a role in controlling various key cellular processes like cell shape changes, initiating proliferation, recruitment of immune cells, calcium ion (Ca^+2^) signaling in the lumen of endoplasmic reticulum and mitochondria-associated membranes [54,84]. It acts as a secondary messenger in insulin signaling and several growth factor-induced signaling cascades [54]. Also, H_2_O_2_ is involved in the chemical modifications of specific Cys amino acids, which are expressed in some cellular proteins [85]. H_2_O_2_ generated during physiological oxidative stress conditions in the concentration of around (1–10 nM) acts as a redox signaling molecule in various cellular processes creating oxidative eustress, although, the higher or pathological concentration of H_2_O_2_ of around (>100 nM) is known to cause deleterious effects to cellular biomolecules, this effect is called oxidative distress (Figure 2) [55]. According to many reports, the higher pathological concentration of H_2_O_2_ in oxidative stress conditions can go up to 150µM [86,87,88].

Various studies have been conducted to examine the concentration at which the H_2_O_2_ acts as a cytotoxic and neurotoxic agent. Further, various studies have investigated the mode of cell death caused by H_2_O_2_, mainly due to apoptosis or necrosis [89,90,91]. It was found that the effects of H_2_O_2_ are largely dependent upon the mode of cell death induced (apoptosis or necrosis) depending on the cell type used, its physiological state, length of exposure to H_2_O_2_, the H_2_O_2_ concentration used, and the cell culture media employed [89,92]. Yoshiro and colleagues (2006) [93], investigated that 50µM of H_2_O_2_ exhibited caspase-9 and caspase-3 activation, finally leading to apoptotic cell death in human T-lymphoma Jurkat cells, whereas a higher concentration of 500 µM caused necrotic death. Teramoto and the group (1999) [94], demonstrated that a lower concentration of 10–100 µM predominantly caused apoptosis, however, a higher concentration of 1–10 mM induced necrosis in human lung fibroblasts cells. Troyano and associates (2003) [95], demonstrated caspase-9 and caspase-3 activation and death by apoptosis in U-937 human promonocytic cells when treated with 200 µM H_2_O_2_. Although, treatment with 2 mM H_2_O_2_ caused necrosis. Gulden and the group (2010) [86], investigated in detail how exposure time and cell concentration affect the cytotoxic potency of H_2_O_2_ in vitro. They investigated those median cytotoxic concentrations decreased from 500 to 30 μM with increasing incubation time from 1 to 24 h. The cytotoxic effects of H_2_O_2_ were also evaluated in neuroblastoma × spinal cord motor neuron cell line (NSC34). A short (30 min) exposure of H_2_O_2_ caused delayed cell death with the median effective concentration (EC_50_) of ~1 mM [96]. Also, treatment of 500 µM H_2_O_2_ for 24 h in a hippocampal neuronal cell line (HT-22) induces around 50% of cell death [97]. Further, investigation of exposure to 1 mM H_2_O_2_ for 2 h on human embryonic kidney 293 cells (HEK293) in vitro, cells displayed the extent of programmed cell death, with Condensed chromatin and apoptotic nuclei [98]. The above-reported studies and various other studies [99,100,101,102,103] elucidated H_2_O_2_ concentration-dependent change in cell signaling and death. Very low concentration of H_2_O_2_ cause cell signaling and hence, cell growth, a mid-higher concentration of around (120 µM to 150 µM) induce a temporary growth arrest, the intermediate concentration of (250 µM–400 µM) causes permanent growth arrest and a higher concentration of (≥1 mM) causes cell damage by necrosis and hence death.

### 3.2. Metabolic Sources and Sinks of Hydrogen Peroxide

**Figure 3:**
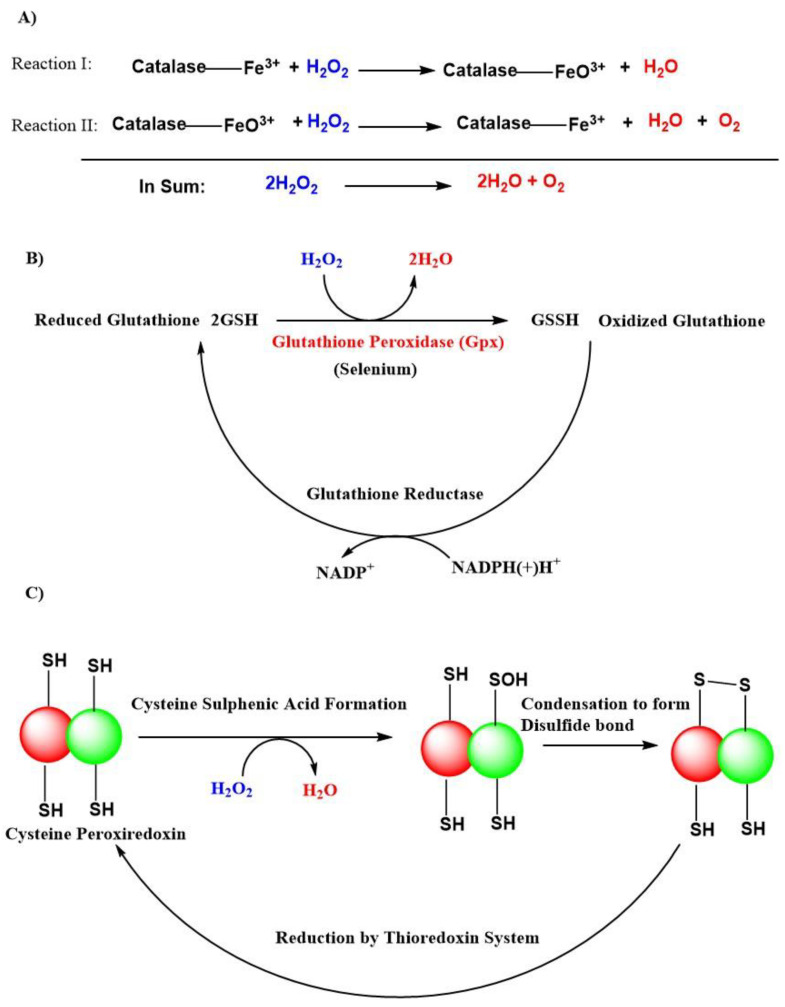
Antioxidant actions of various natural defense systems present in our body to scavenge hydrogen peroxide (H_2_O_2_). (**A**) Catalase system. (**B**) Glutathione peroxidase (GPxs) system. (**C**) Peroxiredoxin system. Abbreviations: (SH), thiol; (-SOH), sulphenic acid; (-S-S-), disulfide bond; NADP+, nicotinamide adenine dinucleotide phosphate; NADPH(+)H+, reduced nicotinamide adenine dinucleotide phosphate; GSH, reduced glutathione; GSSH, oxidized glutathione.

The superoxide anion radical is the precursor of all radicals, and it is generated during the respiratory electron transport chain process complexed with cytochrome I, II, and III or by NAD(P)H oxidases (NOXs) enzymes in the mitochondria [104]. A total of 31 H_2_O_2_ generating oxidases have been reported [105]. SODs like SOD1, SOD2, SOD3, with their presence in cytosolic, mitochondrial matrix, and extracellular locations, respectively are the major sources of H_2_O_2_. Besides that, the endoplasmic reticulum and peroxisomes are also responsible for the production of H_2_O_2_ [55]. There exist an H_2_O_2_ gradient, which is largely associated with their generation in association with respiratory cytochromes like complex III and is associated with the generation of H_2_O_2_ within mitochondrial cristae, whereas, complex I and II contribute H_2_O_2_ within the mitochondrial matrix [106]. The H_2_O_2_ removal from cells is mainly carried out by natural antioxidant enzymes like dismutation of catalases, peroxidases like glutathione peroxidases (GPxs), and peroxiredoxins in the form of H_2_O and O_2_ (Figure 3) [55,107,108]. GPxs are a family of selenoenzymes homologous to selenocysteine, containing mammalian GPx-1, and have a high degree of affinity for H_2_O_2_ in humans. GPx-1 is one of the most expressed and abundant members of the GPx family of enzymes that include an epithelial-specific enzyme that is highly expressed in the intestine (GPx-2); a secreted subtype (GPx-3); and GPx-4, which is widely expressed and differs in its substrate specificity compared to the other family members. Accordingly, GPx-1 is a key selenoenzyme, an enzyme involved in alleviating the detrimental effects of H_2_O_2_ [109,110]. It is present in all cells; found in cytosolic, mitochondrial, and, in some cells, in peroxisomal compartments, GPx-1 can also reduce lipid hydroperoxides and other soluble hydroperoxides. GPx-4 [111] and GPx-7 [112] are also known to scavenge H_2_O_2_ in humans but are not globally expressed compared to GPx-1 [109].

### 3.3. Generation of Highly Unstable and Reactive Free Radical Species, the HO^•^ from H_2_O_2_

Fe is a component of various metalloproteins in living systems and is involved in several critical biochemical processes like oxygen transport through hemoglobin, electron transport during respiration in mitochondria, synthesis, and repair of nucleic acids, metabolism of xenobiotics, essential for oxidation-reduction catalysis and bioenergetics though heme of cytochrome enzymes [113]. Three major classes of Fe-containing proteins facilitate oxygen-based chemistry in living cells: iron–sulfur cluster-containing proteins; heme-containing proteins; and iron-containing enzymes that are devoid of iron–sulfur clusters or heme. The redox ability of Fe makes it an indispensable metal of life, making it a key element in numerous biochemical processes of life [114]. However, the ability to take part in (oxidation, reduction cycle) renders Fe to act as a catalyst in a free state to generate toxic free radicals in oxygen-consuming organisms. For this reason, the circulating Fe is protected in a tightly bound state in form of Fe carrier transferrin, which keeps Fe in its inactive redox state. On the other hand, physiological cells also have some free Fe called labile Fe, which acts to generate free radical H_2_O_2_, for redox signaling. However, any alteration in the normal pool of either bound or labile Fe in a state of redox disbalance will give stimulus to start Fenton chemistry to form one of the most toxic radical HO^•^. Over the last few decades, the role of Fe in neurodegenerative diseases has grabbed everybody’s attention [115,116]. The requirement of Fe in the brain is high because of the high demand for energy [117,118]. Fe is an important component for the synthesis of neurotransmitters and myelin sheath of the neuron [119]. Fe toxicity due to Fe deposition and Fe-related oxidative damage is implicated in various neuropathology such as AD, PD, and ALS [120]. A large number of evidence suggests that Fe is involved in the onset and progression of ALS. Fe load was evident in the spinal cord of ALS patients [121], the motor cortex of ALS patients [122], gray matter from the frontal cortex of ALS patients [123], in the serum of ALS patients [124], and the CSF of ALS patients [125]. Recent evidence has shown that oxidative burst due to Fenton chemistry is implicated in the pathology of ALS [116,126,127,128].

**Figure 4:**
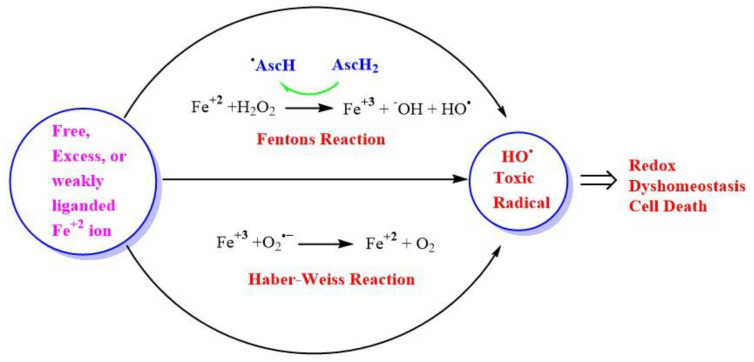
Generation of highly reactive oxidant hydroxyl radicals (HO^•^). Free ferrous ion (Fe^+2^) initiates Fenton reaction with hydrogen peroxide (H_2_O_2_), leading to the generation of a highly reactive hydroxyl free radical (HO^•^). Ascorbic acid (AscH) also, take part in recycling the ferric ion (Fe^+3^), through the generation of (Fe^+2^) and ascorbyl radical (AscH^•^). Superoxide radical anion (O_2_^•−^) can also react with (Fe^+3^) in the Haber-Weiss reaction leading to the production of (Fe^+2^)^,^ which then again starts redox cycling to generate HO^•^. The HO^•^ leads to oxidative stress-induced lipid peroxidation, mitochondrial dysfunction, and an increase in intracellular free-calcium concentration, and finally causing redox imbalance within the cell and ultimately leading to cell death.

Fe and H_2_O_2_ are involved in creating oxidizing environments inside living systems, causing the oxidation of biomolecules and hence cell damage. Being a transition metal, it has the capability to undergo oxidation and reduction inside living systems. Fe can react catalytically with H_2_O_2_ to form highly reactive and toxic species. Higher concentrations of H_2_O_2_ in the range of >100 nM cause disruptive redox signaling, causing oxidative distress and therefore, the oxidation of biomolecules. A higher concentration of H_2_O_2_ undergoes Fenton’s reaction, which is a kind of disproportionation redox chemical reaction in the presence of ferrous ion (Fe^+2^) or Cu. The toxicity of H_2_O_2_ is mainly due to the generation of an OH^•^ via the Fenton reaction in the presence of transition metal ion Fe or Cu (Figure 4), or via Haber–Weiss reaction in the presence of O_2_^•−^ [129].

The OH^•^ is the most powerful oxidant among the ROS, with a potential of E^0^(HO^•^/H_2_O) = 2.34 V. At very low pH, HO^•^ converts into its conjugate base O^•−^ (pKa(HO^•^;O^•−^) = 11.9), the oxide radical, which is less reactive but not relevant at physiological pH [36]. The HO^•^ radical is electrophilic in nature and has a strong affinity towards aromatic and sulfur-containing biomolecules. There are three modes of action for the HO^•^ radical: electron abstraction, hydrogen abstraction, and double bond addition. The addition of HO^•^ radical causes oxidation of biomolecules, like 8-oxoguanine from guanine [130] and 2-oxo-histidine from histidine [131]. Whereas oxidation of sulfur-containing amino acid Met forms Met sulphoxide and sulphone [132]. Electron abstraction is also observed with inorganic substrates like (Fe^+2^) [133]. The H^−^ abstraction mechanism is involved in reactions with various biomolecules, such as polyunsaturated fatty acids like linoleate and arachidonate [134], also from (SH) or hydroxyl (OH) functional groups from different proteins and peptides [135]. This HO^•^ initiates lipid peroxidation to form lipid peroxides ultimately leading to the formation of malonaldehyde or 4-Hydroxy-2-Nonenal (4-HNE), causing alteration in gene expression and promoting cell death [136].

H_2_O_2_ can also be converted into a HO^•^ in the presence of a superoxide radical anion ion called Heber-Weiss reaction [36,137]. Intriguingly, ascorbic acid, which is one of the antioxidants present in the lining of lungs and prevents the harmful effects of pollution, can also generate cytotoxic OH^•^ when it is oxidized in the presence of hydrogen peroxide and transition metal catalyst like Fe and Cu in vitro [71]. This led to the development of acellular assays to measure particle-bound ROS and aerosol oxidative potential (OP) of the particulate matter present in air pollution [138]. Furthermore, it is important to note that, ascorbic acid acts as a pro-oxidant and recycles the ferric ion (Fe^+3^) to (Fe^+2^), hence, facilitating and enhancing the generation of ROS, through successive Fenton cycles [139].

## 4. Amyotrophic Lateral Sclerosis

ALS is a motor neurodegenerative disease (MNDs) that is due to the gradual deterioration of voluntary muscle function due to progressive loss of the lower and upper motor neurons. It is a progressive paralytic disorder, which causes degeneration of motor neurons in the brain and spinal cord [140]. The disease was first described in 1869 by French neurologist Jean-Martin Charcot. Therefore, ALS is also known as Charcot disease in honor of the first person who describes it [140]. The disease become well known in the United States when famous baseball player Lou Gehrig was diagnosed with the disease and died at the age of 37 years [141]. Early symptoms of ALS generally include muscle weakness. Slowly all voluntary muscles are wasted throughout the body, and eventually, the brain loses its ability to control voluntary body movements. Individuals suffering from ALS lose their strength, ability to speak, eat, move, and even breathe although intellect is largely unaffected [141]. Eventually, people with ALS die due to respiratory failure, usually within 3–5 years after first diagnosis [140,142]. ALS is the most common MND and it accounts for 80–90% of all MND cases [143]. There is an increasing number of patients diagnosed with ALS globally. Recent reports have shown that the incidence of ALS is between 0.6 and 3.8 per 100,000 persons-years [144,145,146,147]. Recent studies have reported the prevalence of ALS is between 4.1 and 8.4 per 100,000 persons [147,148,149,150,151]. These two parameters show that the burden of the disease is increasing globally [151]. ALS is characterized by two forms. The most common form of this disease is sALS (90–95%), the etiology of which is still unknown and there is no obvious genetic component associated. The remaining (5–10%) of the cases are fALS, which is due to the association of genetic dominant inheritance factor [141].

ALS is a highly complex, incurable, idiopathic illness, and life-threatening disease. It is thought to be a multifactorial disease, with no one cause being well established to date [152]. In 1993, mutations in the SOD1 gene encoding Cu, Zn SOD1 protein were reported as the first genetic link to fALS [7]. Presently, more than 180 mutations are reported in the SOD1 gene [153]. The most common cause of ALS is a mutation of the SOD1 gene encoding the antioxidant enzyme SOD1 [154]. Because of the high incidence of SOD1 mutations, which accounts for 20–25% of fALS cases, which represents 1 to 2% of all ALS cases, SOD1 is regarded as the most comprehensively studied gene and is one of the prime targets to find therapeutic options for treatment of ALS [155]. fALS which is a mutant SOD1 (mSOD1) induced form of ALS and is almost identical to the late-onset, classical form of ALS called sALS. Both of these forms of ALS are clinically indistinguishable and both share common clinical features, such as the presence of inclusion bodies, motor neuron death, and dysfunction, glial reactivity [156]. The only difference between SOD1-fALS and classic sALS is that individuals with sALS have an average age of onset of 56 years, compared with 46 years for fALS with SOD1 mutations [157]. The variability in the clinical course of disease presentation among fALS patients is seen due to the presence of various genetic mutations in the SOD1 gene. Which is found to be similar in the case of sALS [158]. Research has shown that fALS are mainly due to the mutations of four genes: The chromosome 9 open reading frame 72 (C9ORF72) [159], SOD1 [7], RNA-binding protein fused in the sarcoma (FUS) [160], and TAR DNA binding protein 43 (TDP-43) [161]. A large body of evidence suggested that SOD1 plays an indispensable role in the pathogenicity of both forms of ALS and is a common protein, which is pathologically associated with both forms. In the case of fALS, a mutation in the SOD1 gene called mSOD1 causes misfolding of SOD1 protein. On the other hand, in the case of sALS, non-genetic post-translational modifications (PTMs), such as loss of metal, disruption of quaternary structure, and oxidation of wild-type SOD1 protein by free radicals could cause misfolding of SOD1 protein [11,158]. These aberrant conformation cause wtSOD1 in case of sALS to acquire the same toxic function that are observed for fALS-associated mSOD1variants [88,154,155,158,162]. Moreover, a large number of studies have shown that the misfolding of SOD1 protein is the major culprit in causing the loss of around 70% of spinal cord motor neurons [163,164,165,166,167,168]. In addition, a breakthrough was the discovery of SOD1 transgenic mutant mice like (SOD1^G93A^Tg), (SOD1^G37R^Tg) or (SOD1^G85R^Tg). These mSOD1mice developed a motor neuron disease with many pathological changes reminiscent of human ALS [169] and further increased our understanding of the association of the SOD1 gene in ALS [170].

## 5. Cu, Zn Superoxide Dismutase Enzyme

SOD1 is a highly conserved member of the human SOD family of proteins, which also includes SOD2 and SOD3. All the three proteins are distinct from each other and act as antioxidants by scavenging O_2_^•−^ to H_2_O_2_ [171]. SOD1 is mainly found in the cytosol and inner membrane of the mitochondria [172] and comprises ∼1% of total protein in the cell [173]. SOD2 is found mainly in the mitochondrion matrix. Contrary to SOD1 and SOD2, SOD3 is mostly located outside the cell in the extracellular matrix. Another major difference is that SOD1 is a homodimer while SOD2 and SOD3 are homotetrameric proteins; SOD1 and SOD3 catalyze the dismutation of O2^•−^ through dismutation of Cu^+2^, whereas SOD2 utilizes manganese (Mn) as a redox-active transition metal for dismutation. SOD1 isoform of the protein is mainly involved in the pathology of ALS [158].

The Eukaryotic SOD1 is a stable homodimer [174]. The dimer is held together principally by hydrophobic interactions. Each SOD1 monomer contains two transition metal ions, one Cu and one Zinc (Zn), both of which play an important catalytic and structural role in the enzyme [174].

**Figure 5:**
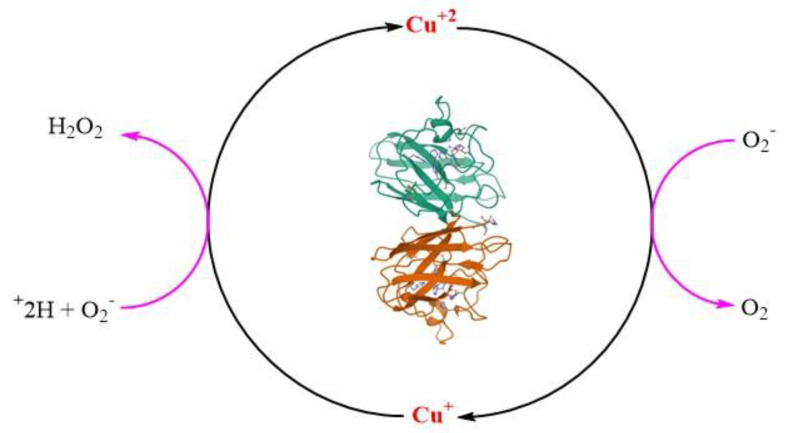
Structure of superoxide dismutase1 (SOD1) dimer (pdb code: 1SPD). The catalytic role of copper (Cu) in the dismutation reaction of superoxide radical anion (O_2_^•−^).

SOD 1 is a 32 kDa homodimeric SOD1 protein and adopts an eight-stranded Greek key beta-barrel structural motif. Homodimerization of SOD1 protein reduces the solvent-accessible area, making it more stable. The fully metallated form of SOD1 protein melts at 85–95 C [175]. Two functional loops are present in SOD1: the electrostatic loop that guides superoxide into the redox-active site where (Cu^+2^) and the Zn-binding loop is located [11,174]. The catalytic Cu is coordinated to SOD1 by four histidines (46, 48, 63 and 120) residues in oxidized form Cu^+2^ and three histidine residues (46, 48 and 120) in its reduced form(Cu^+1^) [176,177]. The structurally vital Zn ion acts as a monodentate ligand and is thought to play an important role in maintaining the structure of SOD1 and acts as a positive charge ion sink. The Zn ion is coordinated by three histidines (63, 71 and 80) and one aspartate (83). Coordination of Cu to SOD1 is required for its catalytic dismutation activity to scavenge O_2_^•−^ [172] through a ping pong mechanism (Figure 5) [11]. Other PTMs like coordination of Zn^2+^, modulating the folding free energy of SOD1 [178], and oxidation of a (-S-S-), are important to form the mature structurally stable SOD1 protein. A distinctive functional feature of SOD1 is the presence of an intra subunit (-S-S-) between Cys^57^ and Cys^146^ (C^57^–C^146^), which is atypical for proteins that reside in the highly reducing environment of the cytosol [174].

## 6. Role of H_2_O_2_ in Misfolding of Cu, Zn SOD1

Even with the above literature findings, we could say that ALS is highly complicated and still the molecular mechanism and its etiology are unknown and not resolved. However, a large body of evidence has shown that SOD1 is the major protein that serves as a hallmark in ALS disease progression. Current research and findings have largely demonstrated two leading hypotheses which are thought to play an indispensable role in ALS disease. Firstly, increased oxidative stress causes SOD1 toxicity, and secondly, aberrant misfolding of SOD1 protein structure. These two factors are interrelated to each other [12]. Before going into the hypothesis of SOD1 misfolding herein, we should discuss what is misfolding of a protein. A protein is said to be folded if it is present in its regular conformation or structure, including the elements of secondary structure [11]. The native state of a protein is often folded and forms the operative structure of a protein. A protein is said to be unfolded if it does not possess a regular structure and is highly soluble [179].

SOD1 maturation or SOD1 folding requires four post-translational maturation steps in addition to N-terminal acetylation. Firstly, Cu insertion, secondly, Zn insertion, thirdly dimerization of monomer units, and lastly, (-S-S-) formation between monomer units. These PTMs provide SOD1 structure, function, and stability. Therefore, any alteration in these PTMs causes accumulation of immature SOD1, causing improper folding or unfolding of SOD1 [11]. It is evident that the maturation of SOD1 to its functional form itself is a universal process that requires oxidation, however, the misfolding of SOD1 that acquires toxic function is due to the result of an oxidative environment of the motor neuron. Several reports have shown that misfolding and toxicity are caused by oxidative free radicals [12,154,180,181]. Therefore, according to our current understanding, herein we aim to contemplate that the most important free radical which is responsible for the misfolding and toxicity of SOD1, is H_2_O_2_, and thereby plays a critical role in the pathophysiology of ALS.

Initial evidence to support our conjecture was given by Liu and co-workers (2020) [115], they have for the first time, using unique microdialysis and microcannula sampling techniques in transgenic mutant mice, demonstrated that the levels of H_2_O_2_ and HO^•^ are significantly higher than the level of O_2_^•−^ in ALS transgenic mutant mice compared to control. Showing the in vivo evidence that the “mSOD1catalyses” the formation of OH^•^ radical through the following steps. In addition, these studies suggested that H_2_O_2_ formed by overexpression of humanSOD1 (hSOD1) is efficiently destroyed, whereas more of H_2_O_2_ produced by the mSOD1 escapes into the tissue where it may produce damaging HO^•^, thereby causing the oxidation of protein, DNA, and membrane phospholipids causing motor degeneration in ALS. Moreover, this study also proves the theory of acquiring a new function of mSOD1, blocking H_2_O_2_ conversion to H_2_O, thereby allowing more HO^•^ formation from H_2_O_2_ [115].

The gain of function theory for mSOD1 in ALS pathogenesis was supported by Yim and co-workers (1990) [23]. They demonstrated the enhancement of free radical formation due to a decrease in the Michaelis constant (Km) for H_2_O_2_ as measured by the spin trapping method in the SOD1^G93A^ ALS model. According to their studies, the free radical generating function of mutant G93A is enhanced compared to the wild type of enzyme, particularly at lower concentrations of H_2_O_2_. This was found to be due to the decrease in the value of Km for H_2_O_2_ for G93A with the same turnover number of the enzyme (Kcat) value for both mSOD1 and wSOD1, thus the ALS symptom observed in the G93A ALS model is due to the gain of function, i.e., an increase in the function to generate free radicals [23]. Thus, this study supported the fact that H_2_O_2_ acts as a substrate to produce an elevated amount of toxic free radicals HO^•^, which is due to the low Km value of H_2_O_2_ for mSOD1 compared to wSOD1. This enhancement of free radical generating function is thought to inactivate the enzyme, through disturbance in the PTMs process to form mature SOD1. Jewett and his team, observed the oxidation of matured SOD1 with H_2_O_2_ causes a sequence of events, the first being the formation of 2-oxo-histidine, Cu loss and thus causing inactivation of the catalytic property of SOD1 [182]. This study again suggested the role of H_2_O_2_ as a substrate in the inactivation of SOD1.

Further, Sampson and co-workers (2001) [183], studied the effects of H_2_O_2_ on Zn deficient SOD (Cu, E SOD) and Cu, Zn SOD using bovine SOD invitro. They demonstrated firstly, that H_2_O_2_ exposure to Cu, E SOD inactivated zinc-binding activity six times faster than dismutase activity. Although, the rate of loss of dismutase activity is the same for both the SOD. Secondly, they detected through UV circular dichroism that H_2_O_2_ instigate elusive changes in the tertiary structure of Cu, E SOD, but not the secondary structure. Finally, they showed that H_2_O_2_ in a lower concentration of 1mM amplifies the toxicity of Zn deficient Cu, E SOD to motor neurons in ALS manifesting the Zn loss from SOD [183]. The aberrant misfolding of mSOD1 adversely affects the binding of Zn in the Zn binding sites of mSOD1, thereby, decreasing the affinity of Zn inmSOD1 [184,185,186]. The gain of the toxic function of SOD1 is due to the loss of Zn metal. Two major factors are involved in the loss of Zn from SOD1 in the case of ALS pathology. The first is the presence of high neurofilaments (NFs), and the second is the presence of a pathological concentration of H_2_O_2._ NFs are intermediate filaments that comprise the neuronal cytoskeleton and are abundant in the axons. Recent evidence has shown the presence of elevated NFs light chain and phosphorylated NFs heavy chain levels in the CSF and serum ALS patients, speculating the extensive damage of motor neurons and axons [187,188,189]. Similar, to SOD1, NFs are found abundantly in motor neurons and are known to bind to metals [190]. In case of elevated levels of NFs in ALS, NFs could act as a sink for Zn and could remove Zn from both wSOD1 and mSOD1, making SOD1 deficient of Zn, thereby, enhance the catalysis of tyrosine nitration by ONOO^−^. Because zinc shares a common histidine ligand with Cu, Zn deficiency may also alter the redox properties of copper [191,192]. Whereas the second factor is the neurotoxic levels of H_2_O_2_ that modifies the (SH) status of both wSOD1 and mSOD1 to acquire the toxic gain of function and thus, causing the elimination of Zn. In the case of increased ROS in motor neurons, the pathological concentration of H_2_O_2_ makes the process of Zn recovery irreversible and thus causing structural defects in SOD1 structure even in the presence of small antioxidants inducing misfolding and thus toxicity to motor neurons [183]. The above findings supported the fact that the pool of Zn in SOD1 is an important factor in the neuropathology of ALS.

The above findings were additionally supported by Rakhit and co-workers (2002) [12], with the help of dynamic light scattering and analytical ultracentrifugation, they found that the most aggregation-prone species is Zn deficient SOD1 and is amenable to form toxic aggregates. As mSOD1 is less stable than wSOD1, oxidative stress through the generation of H_2_O_2_ as substrate causes the formation of an irreversible Zn deficient and consequently a monomeric intermediate state of the protein. These two intermediate acts as a transition state intermediate before aggregation [12]. Thus, this study proved that oxidative stress-induced Zn deficient SOD1 is vulnerable to aggregation and thus misfolding.

Further, studies have identified that the disruption of Cu homeostasis due to oxidative stress is responsible for toxic SOD1 aggregate formation [19,193]. Also, Cu deficiency was found in the spinal cord of transgenic mutant mice having poor locomotor function, supporting the role of Cu in maintaining the integrity and folding of the SOD1 structure [194,195]. Evidence from the literature has shown that pathological H_2_O_2_ attacks the Cu binding histidine [196,197], eventually leading to Cu loss and hence, SOD1 deactivation [182].

The functional role of metals in maintaining the dynamics of SOD1 is well established by the fact that SOD1 monomer, which is metal deficient apoSOD1 species, are vulnerable to change in dynamics leading to misfolding and hence act as a precursor for aggregation. [11,12,198,199,200]. Therefore, both the metals are vital in maintaining the homeostasis and redox properties of SOD1 protein and loss of metals called demetallation due to oxidative stress caused by pathological H_2_O_2_ from holoSOD1 causing its misfolding and hence disease progression of ALS.

The peroxidase hypothesis describes that mSOD1 could act as a peroxidase and has the ability to generate HO^•^ radical from H_2_O_2_, thus creating an oxidative environment in motor neurons creating toxic aggregates [201]. Other studies showed that the biologically ubiquitous bicarbonate buffer-dependent reaction initiated by H_2_O_2_ generated by peroxidase activity of hSOD1 causes the formation of oxidation products of the hSOD1-Trp32 residue, particularly the covalent dimer, in triggering the non-amyloid aggregation of hSOD1 [202]. This study was well supported by other reports, which suggested that the overexpression of hSOD1WT in mice causes ALS [203], and, the presence of over-oxidized/carbonylated hSOD1WT in sporadic ALS patients [204].

**Figure 6:**
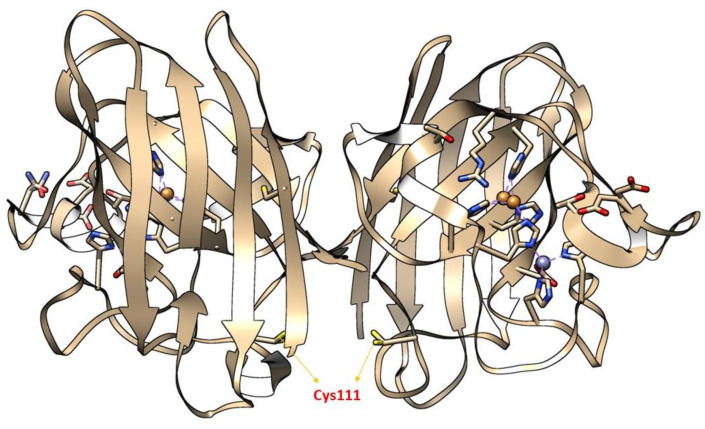
The X-ray crystallographic structure of wild-type SOD1 (wSOD1) (Pdb#2C9V) [211] is shown, modeled in Chimera. Cysteine 111(Cys^111^) highlighted in the yellow act as a “HOT SPOT” for oxidative modification by hydrogen peroxide (H_2_O_2_) and labeled red in the cartoon. The Zinc (Zn) and Copper (Cu) atoms are shown in cyan and orange, respectively [210].

Moreover, the importance of (SH) status in the central nervous system (CNS) also determines the cytotoxic level of H_2_O_2_. During the cellular process, (SH) oxidation mainly of Cys and glutathione act as a hot spot in CNS, to produce neurotoxic H_2_O_2_, thereby making neurons vulnerable to HO^•^ radicals [205]. A large body of evidence has shown that redox modification of a Cys amino acid, mainly Cys^111^ in SOD1, by H_2_O_2_ is mainly implicated in the pathology of ALS [206,207,208,209]. Cys^111^ is a primary target for oxidative modification and plays a crucial role in oxidative damage to hSOD1, including mSOD1 [206] (Figure 6). Bosco and colleagues (2010) [210], employed Fourier transform mass spectrometry (FT-MS) to confirm the oxidation of wSOD1 with the exposure of H_2_O_2_. They found that there was an increase of 48 Da for the predominant species in the spectrum of oxidized wSOD1, compared to unmodified wSOD1. Further, they confirmed with the help of electron capture dissociation (ECD) technique, that the (SH) group in Cys^111^, encoded in exon 4 to be the most vulnerable amino acid, which is found to be irreversibly oxidized to sulphonic acid, through the addition of three atoms of oxygen. Therefore, Cys^111^ acts as a hot spot for oxidative modification by H_2_O_2_ [210]. Supporting the role of H_2_O_2_ in altering the spectroscopic and biophysical properties of Cys^111^ in SOD1 [207].

Kong and group (2012) [208], examined the redox state of SOD1 Cys residues in the G37R transgenic animal model under oxidative stress induced by H_2_O_2._ The data suggested that with the progression of disease there is an increase in oxidation of (SH) groups of Cys residues due to an oxidative burden inside the spinal cord motor neurons. This was further proved by an upper shift band in reducing SDS-PAGE, which turned out to be a Cys^111^-peroxidized SOD1 species using MalPEG. MalPEG is an alkylating agent linked with 5 kDa PEG, easily reacts with sulfhydryl groups of Cys residues, and causes a 5 kDa increase in molecular weight per one modification on SDS-PAGE. They demonstrated that oxidation by H_2_O_2_ decreased the MalPEG modification and increased Cys^111^-peroxidation in G37R spinal cord extract. In addition, they also found the formation of different aggregates and multimers during the disease progression which is thought to be due to the formation of abnormal conformational change of SOD1 due to Cys^111^ modification [208]. Thus, the more reactive thiolate of Cys^111^ is critically involved in the aggregation of the SOD1 process rather than a (-S-S-) causing (-S-S-) independent SOD1 aggregation. Thus, this data supported the role of H_2_O_2_ in the aggregation of SOD1 via the formation of (-S^−^) in Cys amino acid residue. 

Like that of SOD1, TDP-43 is also implicated in the etiology of ALS and this is well supported by many studies [21,212,213,214,215]. Cohen (2012) [212] provided evidence that oxidative stress causes inhibition of TDP-43-mediated RNA regulatory functions. Further, they found that redox disbalance caused by stressors like pathological concentration of H_2_O_2_ (1–10 mM), causes unusual TDP-43 cross-linking via Cys oxidation and (-S-S-) formation leading to decreased TDP-43 solubility and hence, TDP-43 formation of toxic aggregates, implicated in the pathology of ALS. Moreover, the pathological modification is due to the mislocalization of TDP-43 from the nucleus to the cytoplasm. Further, TDP-43 proteinopathies induced by the pathological concentration of H_2_O_2_ were well demonstrated by Chang (2013) [216]. Lin and group (2020) [217] demonstrated the phenomenon of aberrant oxidative modification of sulfur-containing amino acid Met. Above mentioned studies have shown the role of pathological H_2_O_2_ induced TDP-43 proteinopathy in the pathogenesis of ALS [218].

**Figure 7:**
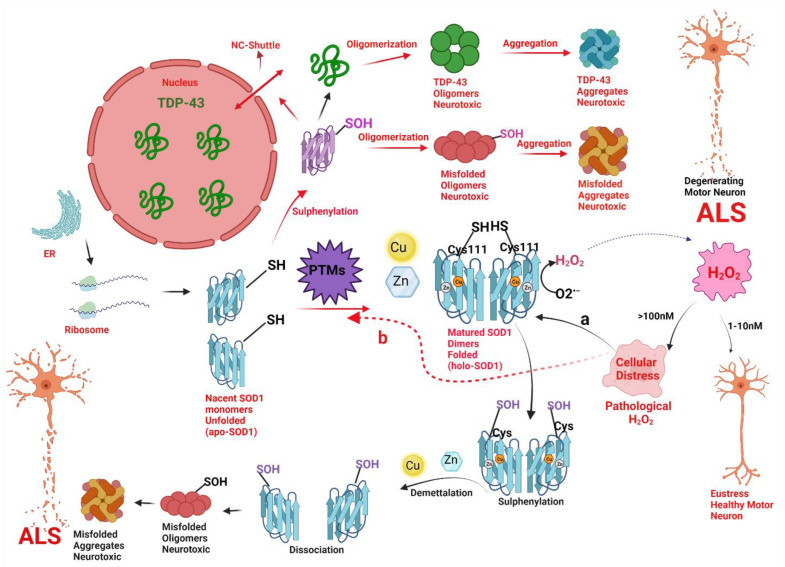
A hypothetical model implicating how pathologic concentrations of hydrogen peroxide (H_2_O_2_) prompt the change in conformation and biophysical properties of superoxide dismutase (SOD1) via change in thiol (SH) status of Cys^111^, and thus instigate SOD1 and TAR DNA-binding protein (TDP-43) toxicity in motor neuronal cells leading to a degeneration of motor neurons in amyotrophic lateral sclerosis (ALS). Nascent SOD1 (unfolded, apoSOD1) released from the ribosomes, undergo post-translational modifications (PTMs) to form homodimerized matured SOD1 (folded, holoSOD1) through the addition of Copper (Cu) and Zinc (Zn) on the correct binding sites of apoSOD1 and formation of disulfide bond (-S-S-). Mature SOD1 catalyzes the dismutation of superoxide radical anion (O_2_^•−^) to H_2_O_2_. H_2_O_2_ acts as a double edge sword molecule and in low concentration i.e., the physiological concentration of (1–10nM) acts as a signal molecule to create eustress in the cell. However, at higher concentrations i.e., the pathological concentration of (>100 nM) acts to create an oxidative environment for the cells to create cellular distress. The pathological concentration of H_2_O_2_ acts as a reactive oxygen species (ROS) and could cause misfolding of SOD1 via two molecular pathways (**Path a** and **path b**).

Cheng Xu and colleagues (2018) [21], explained how the pathological concentration of H_2_O_2_ regulates the redox biology of Cys^111^ and regulates the misfolding and toxicity of SOD1 and TDP-43 associated with ALS and suggested that sulfenic acid modification of wSOD1 play a crucial role in the pathogenesis of sporadic ALS. They demonstrated firstly that the increasing concentration of H_2_O_2_ from 20 to 200 μM increased the concentration of apo-SOD1 filaments by incredibly increasing maximum thioflavin T (ThT) fluorescence intensity. Secondly, they showed that pathological concentrations of 100 μM H_2_O_2_ activate the fibrillization of wild-type human SOD1 in a neuroblastoma cell line (SH-SY5Y). Finally, they detected the sulfenic acid modification of SOD1 via sulfenation of Cys^111^ by pathological concentration of (0–200 μM) H_2_O_2_. Based on the intriguing in vitro experimental data, they continued the experiments to see whether these PTMs could also be observed in cerebrospinal fluid (CSF) of sALS patients. Interestingly, they observed the increased level of sulfenic acid-modified wild-type SOD1 level in cerebrospinal fluid (CSF) of 15 sALS patients compared with 6 age-matched non-ALS control patients. Finally, they hypothesized that pathological concentration of H_2_O_2_ triggers SOD1 fibrillization, by over oxidizing the (SH) of Cys-111. These (-SOH) modified SOD1 is expected to cause cytoplasm mislocalization of human TDP-43 (from the nucleus to the cytosol), forming cytoplasmic TDP-43 oligomers [21]. Thus, change in abnormal (SH) status of Cys^111^ in SOD1 and has been implicated in toxicity of SOD1 and TDP-43 in motor neurons and is illustrated below (Figure 7). These studies once again proved the role of pathological H_2_O_2_ in the pathogenesis of ALS. Further, it also supported the fact that SOD1 aggregates could increase the propensity of TDP-43 aggregation.

**Path a (black arrow):** The pathological concentration of H_2_O_2_ will cause an abnormal change in thiol status of Cys^111^ (from SOD1-SH to SOD1-SOH) from holoSOD1 called sulfenylation and cause the release of metal-bound ligands like Cu and Zn from the active site of holoSOD1, called demetallation. Sulphenylation with demetallation **(path a)** leads to dissociation of homodimerized holoSOD1 to its monomeric metal deficient SOD1, hence, encouraging the oligomerization and aggregation of SOD1 to neurotoxic misfolded oligomers and aggregates of SOD1 respectively. Both the oligomers and aggregates of SOD1 are neurotoxic to motor neurons and are implicated in the pathogenesis of ALS.

**Path b (Red arrow):** The pathological concentration of H_2_O_2_ will cause disruptive post-translational modifications (PTMs) via a change in thiol status of Cys^111^ (from SOD1-SH to SOD1-SOH) called sulphenylation of apoSOD1. Sulphenylation thus provokes the oligomerization and aggregation of SOD1 to neurotoxic misfolded oligomers and aggregates of SOD1 respectively. Sulfenic acid-modified SOD1 oligomers could also disrupt the nucleocytoplasmic shuttle (NS-shuttle) between nucleus and cytoplasm, which is responsible for correct translocation of TDP-43 from the cytoplasm to the nucleus, thus incites the mislocalization of TDP-43 from nucleus to cytoplasm and therefore, provoking the oligomerization and aggregation of TDP-43 to neurotoxic misfolded oligomers and aggregates of TDP-43 respectively. Both the oligomers and aggregates of SOD1 and TDP-43 are neurotoxic to motor neurons and are implicated in the pathogenesis of ALS.

## 7. Conclusions

Oxidative stress due to unstable bio-reactive species is the key factor in the pathophysiology of several neurodegenerative diseases. ALS is one of the highly complex forms of neurodegenerative disease, for which our knowledge about the disease is still scarce and obscure. ALS has complex biology with a large number of variabilities in the molecular targets. However, the discovery of mutations in SOD1 genes encoding SOD1 proteins provided evidence that oxidative stress-induced misfolding of SOD can acquire a toxic gain of function and thereby, act as a critical pathological marker in the pathogenicity of ALS. Literature from the past 20 years has shown that changes in the redox properties of SOD1 cause abnormal conformational changes in the structure of the SOD1 protein leading to a gain of toxic properties, destroying motor neurons in both forms of ALS i.e., sALS and fALS. Therefore, the formation of heterodimers, oligomerization, fibrilization, and toxic misfolded inclusions of SOD1 due to oxidative stress is thought to be common in the etiology of ALS. The alteration in the biophysical properties of ubiquitous native SOD1 protein is caused by the dismutation product of the SOD1 catalyzed reaction, called H_2_O_2_.

H_2_O_2_ can display both Jekyll and Hyde behavior as a stable ‘diffusible’ non-radical oxidant in living cells. The dictation and punctuation of cell signals at various molecular levels depend upon their concentration. Higher concentration of H_2_O_2_, called pathological concentration, acts as a precursor to generating nature’s most reactive, harmful, and toxic species called HO^•^ radical, thus changing the cellular redox thiol status of SOD1 and TDP-43 proteins, and is implicated in the neurodegeneration of motor neurons in ALS. In essence, with our current knowledge, we could say that H_2_O_2_ acts as a modulator of SOD1 redox state mainly via the regulation of PTMs. Hopefully, this insight into the role of H_2_O_2_ in ALS pathology will incite researchers around the globe to think critically about the role of H_2_O_2_ as a decisive denominator in disease progression and may lead to the development of effective therapeutic approaches to stop the devastating course of this disease. To recapitulate, we could say that H_2_O_2_ is the main foci of ROS biology and can act as a principal ROS member in the pathophysiology of ALS.

## Abbreviations

RDH: Redox dyshomeostasis; MND: Motor neurodegenerative disease; ALS: Amyotrophic lateral sclerosis; Cu/Zn SOD, SOD1: Copper/zinc superoxide dismutase; mSOD1: Mutant SOD1; ROS: Reactive oxygen species; sALS: Sporadic ALS; fALS: Familial ALS; SOD1: Superoxide dismutase 1; H_2_O_2_: Hydrogen peroxide; TDP-43: TAR-DNA binding protein 43; NCI: Neuronal cytoplasmic inclusions; AD: Alzheimer’s disease; PD; Parkinson’s disease; HD; Huntington’s disease; (O_2_): Molecular oxygen; Fe: Iron; Cu: Copper; Complex IV: Cytochrome c oxidase; (O_2_^•−^): Superoxide radical anion; (HO^•^): Hydroxyl radical; (Q10): Coenzyme Q; Met: Methionine; Cys: Cysteine; (-SOH): Sulphenic acid; (SH): Sulhydryl or thiol group; (-S-S-): Disulphide bond; (-SO_2_H): Sulfinic acid (-SO_3_H): Sulfonic acid; (NO^•^): Nitric oxide radical; (ONOO^−^): Peroxynitrite; RNS: Reactive nitrogen species; 3-NT: 3-nitrotyrosine; PTMs: Post translational modifications; HOCl: Hypochlorous acid; (^−^OOH): Hydroperoxide nucleophile;(O−O): Peroxide; (-S^−^): Thiolate; (SN2): Bimolecular nucleophilic substitution; (QM-MM): Quantum-classical molecular dynamics simulations; (Ca^+2^): Calcium ion; NOXs: NAD(P)H oxidases; (OH): Hydroxyl; OP: Oxidative potential; 4-HNE: 4-Hydroxy-2-Nonenal; (Fe^+2^): Ferrous ion; (Fe^+3^): Ferric ion; C9ORF72: Chromosome 9 open reading frame 72; FUS: RNA-binding protein fused in Sarcoma; Tg: Transgenic; Zn: Zinc; hSOD1: HumanSOD1; Km: Michaelis constant; Kcat: Turnover number of the enzyme; (Cu, E SOD): Zn deficient SOD; MalPEG: Maleimide-polyethylene glycol; ThT: Thioflavin T; SH-SY5Y: Neuroblastoma cell line; CSF: Cerebro spinal fluid. NS-shuttle: Nucleocytoplasmic shuttle; FT-MS: Fourier-transform mass spectrometry FT-MS; ECD: Electron capture dissociation; CysSOH: Cysteine sulphenic acid; HEK293: Human embryonic kidney 293 cells; NSC34: Neuroblastoma × spinal cord motor neuron cell line; EC_50_: Median effective concentration; DNA: Deoxyribonucleic acid; (NFs): Neurofilaments; (GPxs): Glutathione peroxidases.
